# Immunomodulation exerted by galectins: a land of opportunity in rare cancers

**DOI:** 10.3389/fimmu.2023.1301025

**Published:** 2023-11-08

**Authors:** Laura Díaz-Alvarez, Georgina I. López-Cortés, Erandi Pérez-Figueroa

**Affiliations:** ^1^ Departamento de Inmunología, Instituto de Investigaciones Biomédicas, Universidad Nacional Autónoma de México, Mexico City, Mexico; ^2^ Posgrado en Ciencias Biológicas, Unidad de Posgrado, Universidad Nacional Autónoma de México, Mexico City, Mexico; ^3^ Facultad de Química, Universidad Nacional Autónoma de México, Mexico City, Mexico; ^4^ Unidad Periférica para el Estudio de la Neuroinflamación en Patologías Neurológicas, Instituto de Investigaciones Biomédicas e Instituto Nacional de Neurología y Neurocirugía, Universidad Nacional Autónoma de México, Mexico City, Mexico; ^5^ Departamento de Bioquímica, Facultad de Medicina, Universidad Nacional Autónoma de México, Mexico City, Mexico

**Keywords:** cancer, rare cancer, galectins, immunomodulation, cell signaling, therapeutics

## Abstract

Rare cancers represent only 5% of newly diagnosed malignancies. However, in some cases, they account for up to 50% of the deaths attributed to cancer in their corresponding organ. Part of the reason is that treatment options are generally quite limited, non-specific, and very often, only palliative. Needless to say, research for tailored treatments is warranted. Molecules that exert immunomodulation of the tumor microenvironment are attractive drug targets. One such group is galectins. Thus, in this review we summarize the current knowledge about galectin-mediated immunomodulation in rare cancers, highlighting the research opportunities in each case.

## 1 Introduction

Rare cancers, defined as those affecting a limited number of individuals, present distinct challenges in research and treatment due to their reduced prevalence. In the United States, the National Cancer Institute designates rare cancers as those impacting fewer than 40,000 individuals annually (approximately one in 10,000), while the European Society for Medical Oncology sets the threshold at fewer than 5 individuals per 10,000 (1, 2). Up to date, there are 198 categorized rare cancers (2), encompassing diverse tumor types like bone (e.g., adamantinoma and chordoma), digestive system (e.g., appendiceal cancer and cholangiocarcinoma), and glandular malignancies (e.g., adrenocortical carcinoma and anaplastic thyroid cancer). Due to their infrequent occurrence, currently available therapies often stem from those designed for more common cancers, lacking tailored approaches that consider unique molecular mechanisms. Rare cancer patients often face a higher likelihood of progressing to terminal stages. Consequently, certain rare cancers exhibit alarmingly high mortality rates, like desmoplastic small round cell tumors with an 85% fatality rate (1). In 2022 the American Cancer Society registered over 600,000 cancer-related deaths (3; 4), 25% of which are attributable to rare cancers (1), emphasizing the urgent need for targeted interventions in this realm.

Galectins, a group of evolutionarily conserved proteins in eukaryotes, play a pivotal role in the immunomodulatory landscape of prevalent cancers. Thus, their exploration as potential drug targets holds promise for rare cancer treatments. Comprising 12 human members (5), galectins are characterized by their carbohydrate-recognizing domain (CRD), enabling them to bind to β-galactosides (reviewed in 6). These proteins are classified into three distinct structural groups: prototype, tandem, and chimera. Each group features specific arrangements of CRDs and accompanying protein-binding domains. The prototype group (Galectin-1 [Gal-1], -2, -7, -10, -13, -14, and -16) has a single CRD, the tandem group (Gal-4, -8, -9, and -12) exhibits 2 of such domains, and the chimera group (Gal-3) bears one CRD and a protein-binding domain (5).

Several galectins have been implicated in immunomodulatory functions. For instance, Gal-9 a natural ligand for T cell immunoglobulin mucin 3 (Tim-3), is expressed in both myeloid (monocytes, dendritic cells, and myeloid-derived suppressor cells) and lymphoid immune cell populations (Treg, Th17, and activated Th1 lymphocytes) (7, 8). Tim-3’s overexpression on exhausted T cells and dendritic cells underscores the importance of Gal-9’s interaction in immunosuppressive regulation, a factor significant in both chronic viral infections and cancer contexts (9). Gal-1 and Gal-3 facilitate the epithelial-mesenchymal transition (EMT) (10, 11). Gal-1 also promotes the TGF-β-induced transformation of normal fibroblasts into tumor-associated fibroblasts (TAFs) (12, 13). TAFs further secrete Gal-1 to the microenvironment, which binds β1 integrin, triggering the subsequent expression of Glioma-associated oncogene 1 (Gli1) (10), a pivotal regulator of the Hedgehog signaling pathway governing adult cell division and homeostasis in stem cell subpopulations. Activation of the Hedgehog pathway through Gli1 enhances stem cell-like properties of cancer cells, consequently promoting EMT and metastasis (10). Thus, Gal-1 contributes in different manners to the progression of metastatic cancer. Gal-3 antagonizes INF-γ-induced signal transduction via AKT/GSK-3β/SHP2 in gastric cancer cells (14). Of note, following treatment with cisplatin, a common chemotherapy drug, Gal-3 is upregulated in surviving cells, promoting unresponsiveness to IFN-γ. Similarly, Gal-7 appears to be a key player in the immunosuppression-driven metastasis in squamous cell carcinoma, as recently shown by An et al. (15). They analyzed a culture of lymph node metastatic cells from a squamous cell carcinoma mouse model. Its RNA-seq pattern showed that interferon-related genes were downregulated. In parallel, tumors from the *in vivo* model displayed areas of immunosuppression, characterized by a deficit of anti-tumoral immune cells. Gal-7 was among a group of upregulated genes in those areas. When Gal-7 was deleted from tumorigenic cells, lung, and lymph node metastases were not found, despite no significant differences in primary tumor size as compared to the controls.

Despite a wealth of evidence supporting the vital role of galectins in immune regulation in prevalent cancers, a considerable gap in knowledge remains between common and rare cancers. This review aims to summarize the current knowledge about immune regulation by galectins in rare cancers. We intend to show the vast land of opportunity for researchers who seek to uncover novel therapeutic strategies to address the unique challenges presented by these infrequent malignancies.

## 2 Thyroid carcinomas

### 2.1 Anaplastic thyroid carcinoma (ATC)

Despite being the 9th most diagnosed carcinomas worldwide and, the most common endocrine malignancies, thyroid cancers have a very low death rate (16, 17). Except for ATC, which is so aggressive that the historical median survival is only five months (18). As a result, despite ATC representing only 2% of all thyroid cancers, it accounts for half of the deaths attributed to them: more than 40,000 globally in 2020, most of them women (16, 17, 19). The main reasons for the elevated death rate are that ATC is generally diagnosed at a very late stage, it progresses quite rapidly and there is no focused treatment (20). Hence, the urgent need to explore potentially specific drug targets such as galectins.

Most of the thyroid gland consists of hormone-producing units called follicles, comprised of follicle cells surrounding the colloid, a protein-rich solution (21). Most thyroid tumors, both benign (adenomas) and cancerous (carcinomas), arise from differentiated follicle cells. In contrast. ATC is an undifferentiated tumor and can sometimes progress from differentiated types, being Gal-3 one of its transformation markers (21–24).

#### 2.1.1 Gal-1 in ATC

Gal-1 is linked to proliferation and invasion in ATC *in vitro* (25). Thus, there is an interest in investigating the therapeutic properties of Gal-1 inhibitors. Gheysen et al. (26) used cell lines to highlight the challenges associated with targeting Gal-1 in ATC. They studied the impact of OTX008, an allosteric inhibitor of Gal-1, on ATC cell lines CAL-62 and 8505c. Several *in vitro* parameters related to tumor growth and metastasis, such as cell proliferation, colony formation, cell migration, and cell invasion, were evaluated. CAL-62 cells proved resistant to OTX008 in all the tests, while 8505c cells had different levels of sensitivity to it. These findings underscore the need for further profiling of tumors within the same ATC diagnosis. To gain deeper insights, the researchers analyzed protein and phosphorylation profiles in cells exposed to OTX008 using Reverse Phase Protein Array. Intriguingly, the phosphorylation profiles of proteins in CAL-62 and 8505c cell lines were nearly opposite after exposure to the Gal-1 inhibitor. Moreover, the study revealed potential resistance mechanisms of CAL-62 cell line to OTX008, involving the upregulation of molecules like CREB1, FOXO3, and GATA3, as well as the downregulation of AKT1, PIK3CA, PTPN12, and other factors. One alternative involves the signaling pathways branching from Ras, a well-known Gal-1 ligand. The authors suggest that OTX008 inhibits this pathway in 8505c cells, as evidenced by a decrease in pERK after prolonged low-dose treatment. Additionally, the study shed light on the subcellular localization of Gal-1, which appears to play a role in determining sensitivity to the inhibitor. In basal conditions, Gal-1 was present in both the nucleus and cytoplasm of 8505c cells and expressed at significantly higher levels compared to CAL-62 cells, where it was predominantly found in the cytoplasm. These findings align with previous reports indicating that the majority, though not all, of ATC samples, exhibit elevated levels of Gal-1 mRNA compared to normal or hyperplastic thyroid tissue (27). However, high-dose treatment with OTX008 resulted in a strong decrease of Gal-1 in the cytoplasm of 8505c cells, while its levels increased in both the cytoplasm and nucleus of CAL-62 cells. This indicates that not only the expression levels of Gal-1 but also its subcellular localization during treatment, are crucial in determining sensitivity to the inhibitor. Finally, the researchers assessed the effects of OTX008 in an *in vivo* setting, which mirrored the *in vitro* findings. In a xenograft mouse model injected with the 8505c cell line, both tumor growth and metastasis were reduced upon treatment with OTX008.

#### 2.1.2 Gal-3 in ATC

The expression of Gal-3 in ATC cell lines 8505c and 8305c is related to the runx2 transcription factor (28). This transcription factor also governs the expression of MMP2 and 9, which confer invasive potential to cancer cells. At the epigenetic level, the Gal-3 gene exhibits reduced methylation in five CpG sites in thyroid cancers compared to healthy tissue (29). However, the presence of Gal-3 in ATC lesions from patients is far from established due to the vast existence of conflicting data.

For more than 20 years, Gal-3 has been recognized as a marker of differentiated thyroid carcinoma (30), especially follicular carcinoma and, particularly the minimally invasive form (31). In contrast, a number of groups have reported the absence of Gal-3 from patient ATC samples (32–34). For example, Rossi et al. examined a group of 34 thyroid cancer samples where they clearly observed that all differentiated and poorly differentiated tumors expressed Gal-3 (35). Whereas all nine ATC tumor specimens were negative for Gal-3 upon immunohistochemical testing.

An extra layer of complexity in this malignancy is that differentiated and anaplastic carcinoma can coexist (33), most likely because the latter can arise from the former (36). Gal-3 seems to play an important role in this transition, as Selemetjev et al. reported in 2015 (23). They analyzed a large set of thyroid cancer samples, both differentiated (Papillary carcinoma of the thyroid, n=69) and ATC (n=30) by immunohistochemistry, and found that Gal-3 levels decrease when papillary carcinoma lesions progress to ATC. This happens simultaneously with an increase in the antiapoptotic protein survivin, partly explaining why ATC is so resistant to treatment. In contrast, Park et al. (22) reported the need for the co-expression of Gal-3, p53, and Ki-67 for the differentiated tumor to adopt anaplastic characteristics.

Thus, early on there were some reports that suggested the presence of Gal-3 in ATC lesions albeit at lower levels than in differentiated tumors (37). In 1999 the group of Fernández et al. (38) showed that all five ATC samples, out of a total of 41 thyroid cancer specimens they examined, expressed Gal-3. Other research groups reported similar findings (39, 40).Gal-3 is also expressed in certain ATC cell lines like FRO82-1, CAL62, ARO, and ACT1 cell lines (41–43).

Therefore, it is clear that the expression of Gal-3 is not homogeneous among ATC cells (42, 44), leading to conflicting statements regarding its usefulness as an indicator of aggressiveness or even as a diagnostic marker (39, 45, 46).

In healthy cells, p53 induces apoptosis by downregulating Gal-3, Lavra et al. demonstrated that the p53 gain-of-function mutant R273H enables the ATC ARO cell line to constitutively express Gal-3, increasing its chemoresistance (41). Gal-3 also promotes the chronic activation of K-ras (47), a phenomenon related to proliferation and tumor growth. This partly explains the results of Menachem et al, who observed that anti-Gal 3 treatment inhibits tumor growth in an ATC mouse model (48). Furthermore, Zheng et al. described that HIF-1α-induced upregulation of Gal-3 in the ATC cell line 8305c promotes cell migration in a hypoxic milieu. Knocking down of Gal-3 inhibits the signaling pathways where Wnt, MAPK, Src, and Rho participate (49), potentially reducing cell motility during hypoxia. Of note, Ras and p53 are up and downstream HIF-1α, respectively. Thus, selective inhibition of Gal-3 in ATC is an avenue worth exploring.

#### 2.1.3 Gal-9 in ATC

It is widely known that Gal-9 contributes to the maintenance of an immunosuppressive tumor microenvironment via a number of mechanisms. Recently Pan et al. (50) have dissected the differential gene expression of tumor-associated macrophages in ATC (ATAMs) vs normal thyroid-associated macrophages. Notably, the subpopulation of IL2RA+ VSIG4 + ATAMs presents a hybrid M1/M2 signature, that is, they express markers related to both activation and inhibition of the immune response. Examples of the former are antigen-presenting genes and functions, as for the latter, Gal-9 is mentioned as one of the key molecules driving immunosuppression. Gal-9 interacts with its receptor TIM-3 on the surface of T lymphocytes. CD8+ lymphocytes respond by weakening their cytotoxic activity, whereas Tregs are activated upon the encounter with Gal-9. Thus, the presence of IL2RA+ VSIG4 + ATAMs correlates with high lymphocyte infiltration (B, CD8+, and Tregs) as well as BRAF and Ras signaling in the tumor. However, despite the elevated number of CD8+ T cells, a great proportion of them are exhausted due to the inhibitory signals released by ATAMs, including Gal-9. Thus, considering the counterintuitive association of IL2RA+ VSIG4 + ATAMs infiltration with favorable prognosis, if immunosuppressive molecules could be specifically targeted in this population, it could skew their phenotype to an M1, further helping eliminate the tumor. This is supported by the findings of Stempin et al. (51), who demonstrated that 8505c-derived conditioned media induces the expression of TIM-3 and the differentiation of THP-1 monocytic cells into M2 macrophages, and the subsequent upregulation of M2 polarization markers CLEC7A, CCL13, and IL6. Incubating the THP-1 monocytes in conditioned media and an anti-TIM-3 antibody significantly reduces the upregulation of M2 markers. This further points to the role of Gal-9 in the M2 skewing of ATAMs, and its potential as a drug target.

### 2.2 Medullary thyroid carcinoma (MTC)

An uncommon neuroendocrine tumor that develops from the neural crest-derived parafollicular C-cells of the thyroid gland is the MTC. MTC accounts for 3-10% of all thyroid cancer cases and is caused by the multiple endocrine neoplasia type 2 syndrome (MEN 2) (52, 53). MEN2 occurs as a consequence of autosomal dominant mutations, gain-of-function like in the rearranged during transfection (RET) proto-oncogene. However reported cases presenting different mutated genes, make more complex the etiology of the cancer. This is why all hereditary and around 40-50% of sporadic cases are related to mutations in RET (54). There have been reported more than 100 different mutations in RET that lead to MTC (52). The relevance of RET lies in its involvement in the development of a variety of organs, including the urogenital system, neural crest, neural ganglia, adrenal medulla, enteric ganglia, and thyroid C cells (52). Not surprisingly, patients with hereditary MEN2 syndrome present at an early age.

Until now the only approved treatments for MTC are radiotherapy, surgical resection, RET inhibitors (e.g., selpercatinib and pralsetinib), or targeting downstream proteins in the RET-mediated signaling, such as RAS (54). RET is a tyrosine kinase transmembrane receptor that regulates a wide variety of cellular processes including survival, proliferation, motility, and apoptosis (54, 55). As mentioned before, different mutations cause a gain-of-function, for example, there are single mutations in exon 16 that cause conformational change in the intracellular domain allowing constitutive kinase activation (52). After RET is activated, the signaling through PI3K/AKT/mTOR and RAS/MAPK enhances the cellular process indicated. A promising immune-based therapy is the one with an anti-PDL-1 antibody, since MTC cells express the inhibitory receptor, impeding the T cell-mediated response.

Physical examination, neck ultrasonography, and cytology are used to diagnose MTC. Also, elevated serum calcitonin and carcinoembryonic antigen (CEA) levels are used as cancer markers (54, 56). Calcitonin levels are explained by the aberrant growth of the parafollicular C-cells of the thyroid gland, which secrete it (52). Additionally, CEA and Gal-3 expression throughout the tumor tissue has been reported on histological patients’ samples with sporadic MTC, and furthermore, the correlation rises in patients with metastatic processes (57, 58). This interaction has been also reported for *in vitro* models of colorectal cancer, where it promotes migration and is suggested to promote metastases (59). So, it is highly possible that CEA and Gal-3 co-expression in the tumor microenvironment enhance the tumor progression and poor survival in MTC patients too. To note, solid cell nests and C cells do co-express CEA and calcitonin, but the Gal-3 expression is determinant for the accurate diagnosis of MTC (60). Therefore, a promising treatment might involve the Gal-3 inhibition to deaccelerate the tumor progression and to reduce the elevated levels of CEA and calcitonin.

## 3 Cholangiocarcinoma (CCA)

The global landscape of liver cancer is characterized by striking variations in prevalence, with developing nations like China accounting for a substantial 55% of cases. In contrast, the rare malignancy CCA exhibits a prevalence of fewer than 6 cases per 100,000 individuals worldwide. A remarkable exception is found in northeast Thailand, where CCA prevalence soars to 110 cases per 100,000 people (61), predominantly attributed to endemic liver fluke *Opisthorchis viverrini* infection. This parasite triggers liver and bile duct obstruction, paving the path to CCA development (62). In the case of Western countries, risk factors for developing CCA are sclerosing cholangitis, hepatolithiasis, and choledochal cysts (63).

In fact, CCA is a group of hepatobiliary cancers affecting the tubes connecting the liver to the gall bladder and then, to the small intestine. Under physiological conditions, these tubes carry the bile necessary for digestion. Given that any tube cell type can give rise to cancer, specific names for the CCA depend on where the malignancy develops. It is called intrahepatic when it affects the bile ducts that are still in the liver, whereas extrahepatic CCA refers to tumors located either in the bile ducts near the intestine (distal) or the gall bladder (hilar). Due to these different cellular origins, the etiology of CCA has not been fully described. However, all the subtypes share a common association with TAFs, which drive rapid disease progression (61). Mostly, CCA develops under chronic inflammatory conditions where cytokines and growth factors promote proliferation and disrupt apoptosis. Specifically, IL-6 increases the expression of myeloid cell leukemia sequence 1, which constitutively activates the STAT/Akt signal transduction pathway. The immediate consequence is the inhibition of apoptosis and the promotion of proliferation (61). Additionally, the production of reactive oxygen and nitrogen species (ROS and NOS, respectively) contributes to DNA damage, a critical factor in cancer development.

The high mortality rate of CCA is rooted in its resistance to anti-cancer drugs (63, 64). Furthermore, some tumors are irresectable, and in the ones that this is possible, patients frequently present local recurrence or metastasis (64). Single-agent or combination chemotherapies (e.g., 5-Fluororuacil (5-FU), cisplatin, and epirubicin) have been used with no significant results (64). Therefore, it is urgent to develop therapies that effectively eliminate cancer cells and ameliorate the prognosis. So far, Thailand’s strategy has been the implementation of control and prevention campaigns for the *O. viverrini* infection (65). In this pursuit, understanding the intricate molecular mechanisms underlying CCA’s behavior is crucial.

### 3.1 Gal-3 in cholangiocarcinoma

Gal-3, an overexpressed protein in CCA, contributes to the multidrug resistance observed in this malignancy by conferring anti-apoptotic properties. Wongkham et al. reported that Gal-3 expression levels in CCA cells correlate with apoptotic resistance to UV radiation, hypoxia, and the anti-cancer drugs 5-FU and cisplatin (63). This resistance stems from Gal-3’s interaction with the Bcl-2-associated death protein (Bad) via an anti-death motif, preventing Bad’s association with mitochondria and blocking apoptosis initiation. Gal-3-deficient cells, in contrast, exhibit apoptosis upon chemotherapeutic stimuli due to a destabilizing interaction of Bad with the mitochondrial membrane, which prompts the release of Cytochrome c, leading to Caspase-3 activation and apoptosis induction (66). Moreover, intranuclear Gal-3 levels are associated with a worse CCA prognosis (67).

Gal-3 inhibition is desirable as a pre-treatment for individuals who will undergo chemotherapy since it reduces drug resistance. Some galectins, like Gal-3, have been found to be inhibited by a variety of small compounds, monoclonal antibodies, peptides, polysaccharides, and other shortened galectins (68, 69). In addition, due to the multifunctionality of galectins, it is highly likely that treatments could be more effective if inhibitors could be targeted to the appropriate subcellular compartment, where galectin levels differ from those of healthy tissues. Research on this objective is beginning to take place. For example, Stegmayr et al. evaluated the polar surface area of small Gal-3 inhibitors to route them to the various cellular compartments (68).

### 3.2 Gal-9 in cholangiocarcinoma

In stark contrast, Gal-9 exerts an opposite effect on CCA tumors, as it inhibits proliferation and induces apoptosis. Thus, demonstrating a potential for therapeutic intervention. Exogenous Gal-9 induces apoptosis by increasing both the Caspase-cleaved keratin 18 and Cytochrome c levels (70). Additionally, Gal-9 decreases signaling from key receptors, including the Epidermal Growth Factor Receptor, the Insulin-like Growth Factor-1 Receptor, the Hepatocyte Growth Factor Receptor, and the Fibroblast Growth Factor Receptor 3 in CCA, resulting in reduced tumor size in CCA cell lines and animal models. This evidence supports the proposal of the Gal-9 administration as a strategy to counteract multidrug-resistant CCA, potentially revolutionizing treatment outcomes for this challenging malignancy.

## 4 Hereditable gastric cancers (HGCs)

Within the spectrum of gastric cancers (GCs), a subset is attributed to hereditary factors, accounting for approximately 10% of all cases. This subgroup encompasses three distinct entities: hereditary diffuse gastric cancer (HDGC), gastric adenocarcinoma and proximal polyposis of the stomach (GAPPS), and familial intestinal gastric cancer (FIGC). Only 1-3% of the cases are associated with a known heritable mutation that increases susceptibility (71). HDGC is associated with mutations in the Cadherin E gene and, in a minor frequency to the Catenin alpha 1 gene; GAPPS is associated with a mutation in the promoter 1B of the Adenomatous polyposis coli (APC) gene which is implicated in the regulation of the cell cycle. Worth noting, APC mutations are risk factors for a cluster of familial adenomatous polyposis, which is a group of cancers with an autosomal dominant component; where polyps might grow in the colon, rectum, and stomach (72). The genetic cause of FIGC still remains unknown (73, 74).

Hereditable GCs have a poor prognosis and, so far, the treatment options are very limited. Currently, the only drugs approved for these malignancies are docetaxel, trastuzumab, and ramucirumab. Detection at an early stage remains a challenge, leaving prophylactic endoscopy and gastrectomy as recommended options for those with heightened susceptibility. However, gastrectomy comes with significant risks, including a 1% mortality rate (71). Thus, due to the limited number of cases and the difficulty of early detection, little is known about the role of galectins in heritable GCs. Hence, we aim to highlight the importance of clarifying the roles of galectins in each cancer subtype rather.

### 4.1 Gal-1 in HGC

Targeting the transcriptional factor Zinc Finger Protein 64, which regulates Gal-1 expression, presents a novel strategy against GCs. Increased intracellular Gal-1 levels activate the MAPK and PI3K/AKT pathways, conferring cancer cells stem cell-like properties. These cells, characterized by limited endocytosis capacity, pose challenges for drug uptake, potentially leading to drug resistance. Moreover, secreted Gal-1 promotes immunosuppression via a T-cell-mediated mechanism.

The marker Forkhead box P3 (FoxP3), typically associated with Tregs, is also expressed by certain cancer cells (75). Tregs, known to contribute to immune tolerance through TGF-β production, play a role in the GC microenvironment. Yoshii et al. demonstrated that TGF-β augmented FoxP3 expression in GC cells, leading to upregulated Indoleamine-2,3-dioxygenase and Gal-1 expression, further enhancing immunosuppression (76). Thus, inhibiting Gal-1 synthesis emerges as a prospective approach to circumvent drug resistance and bolster the immune response (77).

### 4.2 Gal-3 in HGC

Gal-3 is a key factor in the different tumor microenvironments in the diverse GCs as it correlates with reduced overall survival (78). In fact, its secretion is induced by cisplatin, counterproductively promoting tumor growth. Gal-3 enhances cell proliferation, invasion, and migration through the Ral A/Ral BP complex, activating the oncogene c-Myc (79). Additionally in GC and in hepatocellular carcinoma, Gal-3 activates GSK-3β, β-catenin, and the Insulin-like growth factor-binding protein 3; resulting in angiogenesis and EMT (80). Nonetheless, opposing findings suggest varying molecular dysregulations influence these pathways. For example, in the gastric adenocarcinoma cell line AGS, GSK-3β is decreased, and the AKT signal transduction pathway is activated (14). Hence, additional layers of complexity in the cellular dynamics should be considered when studying the role of galectins in cancer and its possible use as a therapeutic option or target.

### 4.3 Gal-7 in HGC

Gal-7 is overexpressed in the AGS cell line. Therefore, the difference in the expression of the diverse galectins may influence the overall balance of signal pathways that are activated in different tumors. A report by Kim et al. showed that Gal-7 overexpression in two GC cell lines hampers cell proliferation, invasion, and migration, meanwhile, ablation of Gal-7 in KATO III cells increases the same cellular functions. Moreover, *in vivo*, Gal-7 overexpression renders human tumoral xenografts incapable of growing in mice (81). They also showed that Gal-7 is downregulated in cancerous tissues of patients compared to their non-cancerous tissues. In fact, those tissues with low levels of Gal-7 had hypermethylated CpG islands at +1566 bp of exon 2 of the Gal-7 gene (81). To sum up, the evidence supports that Gal-7 has potential use as therapy.

### 4.4 Gal-9 in HGC

The effect of Gal-9 on GC patients has been described by Wang et al. Their study revealed a correlation between tumor-associated monocytes expressing upregulated Tim-3 and disease progression. Gal-9 stimulation through Tim-3 led to the secretion of interleukins (IL-6, IL-8, and IL-10) in the tumor microenvironment (8). The immune microenvironment during GC progression remains largely unknown. Petersen et al. identified that GC cell lines exposed to X-irradiation and chemotherapeutic agents 5-FU or doxorubicin exhibited increased extracellular calreticulin, an “eat me” signal. However, in parallel those cells upregulated Gal-9 and PD-1L, reducing the efficacy of the immune response (82). Thus, they tested a chemoradiation treatment followed by either anti-PD-L1 or anti-Gal-9 blocking antibodies and showed that the former induces a more potent immunogenic cell death response. In sheer contrast, Takano et al. found that Gal-9 treatment increases caspase-cleaved keratin 18 levels and reduces VEGFR-3 phosphorylation and IGF-1R in GC cell line, inducing apoptosis and reducing proliferation, respectively (83). Thus, the Gal-9 mechanism of action needs to be further dissected to understand its role in hereditable GCs with different genetic backgrounds and to prescribe an appropriate treatment.

In conclusion, hereditary GC poses a challenging clinical scenario with limited therapeutic options. The intricate interplay of Gal-3, Gal-7, and Gal-9, along with transcriptional factors like Zinc Finger Protein 64, has been unveiled, shedding light on potential targets for intervention. Understanding the complex immune microenvironment and the role of FoxP3 provides further insight into disease progression and potential avenues for therapy. As research continues, uncovering the intricate mechanisms underlying hereditable gastric cancers holds promise for developing novel and effective treatment strategies.

## 5 Chordomas

Chordomas represent a category of malignant neoplasms primarily localized within the spinal column or the cranial base, featuring distinctive molecular alterations associated with notochordal differentiation (84). These tumors are characterized by infiltration and destruction of the surrounding bone and soft tissue and a high locoregional recurrence rate. They also exhibit marked resistance to conventional therapy, including chemotherapy and radiotherapy. The overall survival rate for chordoma patients is approximately 65%. These malignancies are divided into three histological subtypes: classical or conventional, chondroid, and dedifferentiated (85).

Several large population-based studies have estimated the overall incidence rate of chordomas to be 0.08 per 100,000 persons per year (86). While chordomas can affect individuals of all age groups, they are most commonly diagnosed in adulthood, typically occurring between the fifth and sixth decades of life. Incidence in children and adolescents is relatively rare, accounting for less than 5% of all chordoma cases (87). Skull base chordomas, a subset of these malignancies, are particularly uncommon, constituting less than 0.2% of all intracranial neoplasms (88).

Little is known about the genetic events responsible for driving the growth of chordomas, but it is well established that dysregulation of different signaling pathways as canonical as BMP4/SMAD and RAS/RAF/MEK/ERK are involved in the development of these tumors, while others like PI3K/AKT/mTOR are starting to be explored in this context (89–93). Thus, agents that modulate these signaling pathways could become attractive prospects for anticancer therapy. For instance, certain tyrosine kinase inhibitors have shown activity against chordomas by selectively inhibiting intersecting molecular pathways (94). One such pathway is that of mTOR, which is frequently hyperactivated in chordomas and has been suggested as a possible therapeutic target (95, 96). This emanates from the fact that a partial or complete Phosphatase and Tensin Homolog (PTEN) deficiency generates hyperactivation of the Akt/mTOR pathway, therefore PTEN is a negative regulator of this pathway, and its absence contributes to chordoma development (97).

Inhibitors targeting the mTOR pathway are currently scrutinized in phase I/II clinical trials for the treatment of other tumors, like chondrosarcoma (98). Unfortunately, most of them have failed to demonstrate a clinical benefit in terms of disease progression or outcomes, partially due to limitations in sample size and follow-up duration. Hence, the importance of *in vitro* models for the study of the molecular features of chordomas cannot be overstated.

### 5.1 Gal-3 and Gal-9 in chordomas

The CH3 cell line is derived from a primary skull base chordoma with both conventional and chondroid features. These cells do not express PTEN, which contributes to cell proliferation and invasiveness (97). Aside from its effects on the activation of the Akt/mTOR pathway (97), the partial or complete PTEN deficiency potentially induces the secretion of Gal-9 (99), as it does in glioblastoma cells via the AKT-GSK-3β-IRF1 pathway. This is of paramount importance because secreted Gal-9 drives macrophage M2 polarization via the activation of its receptor TIM3, leading to the release of VEGF-A, which stimulates angiogenesis, and supports cell proliferation (100).

Gal-9 and Gal-3 expression is related to both the tumor proliferation index (93, 101), and the invasion capacity of chordomas (102). However, their signaling mechanism has not been described. Nevertheless, some speculation can be made based on the available information on other tumors. For example, in both hepatocellular carcinoma and meningioma Gal-3 activates the PI3K-Akt-GSK-3β-β-catenin signaling cascade by enhancing GSK-3β phosphorylation via the activation of the PI3K-Akt signaling pathway. As a result, β-catenin increases accumulation in the nucleus, regulating angiogenesis and EMT, (80, 103). Similarly, in non-small cell lung cancer Gal-3 regulates stem cell proliferation by regulating β-catenin and EGFR; and promotes angiogenesis through the interaction of both Notch and TLR4 signaling pathways (104).

As mentioned above Gal-9 is involved in the proliferation of tumor cells in chordoma, this may be due to its interaction with molecules such as the programmed cell death ligand-1 (PDL-1), and the indoleamine 2,3-dioxygenase (IDO-1), which are important checkpoints in this cancer (93). The PD-1/PD-L1 pathway is one of the mechanisms whereby the tumor cell can evade the host anti-tumor immune surveillance (105, 106). Of note, there is evidence indicating that the overexpression of PD-L1 in chordoma patients is associated with advanced stages and poor survival (107). Moreover, the overexpression of Gal-9 in these patients has partnered with infiltrating lymphocytes into tumors (102). Extracellular Gal-9 may play a role in immunosurveillance through its interaction with TIM3 on the surface of T lymphocytes, a phenomenon that has been shown in gliomas, osteosarcomas, and gastrointestinal tumors (108–110).

The employment of antibodies or pharmacological agents directed against Gal-3 and Gal-9 presents a potential therapeutic approach for the modulation of proliferation and invasiveness in chordoma cells. Noteworthy, there are no current studies exploring galectins as therapeutic strategies in this type of cancer. However, highly specific anti-Gal-3 antibodies have shown promising results in ovarian cancer (111). Additionally, Gal-3 antagonists such as belapectin (also known as GR-MD-02) have demonstrated the ability to increase effector memory T-cell activation in patients with metastatic melanoma (112). There is also evidence that anti-Gal-9 antibodies promote the expansion of cytotoxic CD8+ cells in a mamary tumor mouse model, reducing a population of tumor-enabling Treg cells (CD4+, Foxp3+, CD25+) (113). Thus, blocking Gal-9 has the potential to enhance immunosurveillance and tumor cell destruction. Furthermore, an alternative avenue for the regulation of proliferation in chordoma cells involves the direct targeting of the mTOR pathway, a notable feature of which is the absence of PTEN. The mTOR pathway can be effectively suppressed using molecules such as the rapamycin analogs deforolimus, everolimus, and temsirolimus, which are undergoing clinical trials for the treatment of various cancers, either as standalone treatments or in combination with inhibitors of other pathways (114).

## 6 Conclusions

The role of galectins in rare cancers is not yet fully understood and there is still plenty of contradicting evidence. Some reports correlate with the expression levels of certain galectins with a good prognosis, while others link the role of galectins with cell survival and proliferation. There is still little evidence showing how galectins could modulate the immune system in the tumor microenvironment in rare cancers which is why we call it a land of opportunity.

It is critical that the study of galectins in cancer microenvironments incorporates information such as its glycan-independent interaction with chemokines, which alters the functions of galectins. For example, the binding of Gal-1 to the platelet Factor 4 (CXCL4) modifies the structural properties of Gal-1 at its carbohydrate binding site (115). Additionally, this interaction increases apoptosis in activated T CD8+ cells but not in resting T CD8+ or CD4+ cells. The same report demonstrates the opposite effect for the interaction between Gal-9 and CCL5, where there is a reduction in Gal-9-induced apoptosis in T CD4+ but not in T CD8+ cells (115). Therefore, not only the molecules present in the microenvironment must be considered, but also their interactions and effects on immune cells.

The infrequency of rare cancers hampers comprehensive study and treatment development. However, currently, there is a vast array of tools that can be used to understand their physiopathology and, particularly, to assess the role of galectins in each one. In silico simulations could predict the behavior of the molecular components of the system in the presence/absence of galectins or other molecules. This approach could also deepen and scale up the comparison with more frequent cancers of similar etiology and be used to propose finely-tuned treatments.

## Author contributions

LD: Conceptualization, Data curation, Investigation, Project administration, Resources, Supervision, Writing – original draft, Writing – review & editing. GL Conceptualization, Data curation, Investigation, Project administration, Resources, Supervision, Writing – original draft, Writing – review & editing. EP: Conceptualization, Data curation, Investigation, Project administration, Resources, Supervision, Writing – original draft, Writing – review & editing.
